# Ultrasonic-assisted extraction of total flavonoids from *Zanthoxylum bungeanum* residue and their allelopathic mechanism on *Microcystis aeruginosa*

**DOI:** 10.1038/s41598-024-64129-x

**Published:** 2024-06-08

**Authors:** Jie Cheng, Chengshuai Xu, Yang Sun, Qiuhan Yu, Shuo Ding, Yucai Wang, Wenxue Wei, Wei Xu, Chaobo Zhang, Donghui Gong

**Affiliations:** 1 School of Life Sciences, Liaocheng University, Liaocheng, 252000 China; 2 School of Life Science and Technology, Inner Mongolia University of Science and Technology, Baotou, 014010 China; 3 Shandong Sanduha Ecological Agriculture Technology Co., Ltd, Liaocheng, 252000 China; 4 Shandong Nongmanyi Agricultural Technology Co., Ltd, Liaocheng, 252000 China

**Keywords:** *Zanthoxylum bungeanum* residue, Total flavonoids, Allelopathic mechanism, Chlorophyll fluorescence, *Microcystis aeruginosa*, Ecology, Limnology, Ocean sciences

## Abstract

Water eutrophication has emerged as a pressing concern for massive algal blooms, and these harmful blooms can potentially generate harmful toxins, which can detrimentally impact the aquatic environment and human health. Consequently, it is imperative to identify a safe and efficient approach to combat algal blooms to safeguard the ecological safety of water. This study aimed to investigate the procedure for extracting total flavonoids from *Z. bungeanum* residue and assess its antioxidant properties. The most favorable parameters for extracting total flavonoids from *Z. bungeanum* residue were a liquid–solid ratio (LSR) of 20 mL/g, a solvent concentration of 60%, an extraction period of 55 min, and an ultrasonic temperature of 80 °C. Meanwhile, the photosynthetic inhibitory mechanism of *Z. bungeanum* residue extracts against *M. aeruginosa* was assessed with a particular focus on the concentration-dependent toxicity effect. *Z. bungeanum* residue extracts damaged the oxygen-evolving complex structure, influenced energy capture and distribution, and inhibited the electron transport of PSII in *M. aeruginosa*. Furthermore, the enhanced capacity for ROS detoxification enables treated cells to sustain their photosynthetic activity. The findings of this study hold considerable relevance for the ecological management community and offer potential avenues for the practical utilization of resources in controlling algal blooms.

## Introduction

Excess nitrogen and phosphorus nutrients gradually enter the water environment under increasing urbanization, resulting in water eutrophication and water quality problems^1^. Water eutrophication has emerged as a pressing concern for massive algal blooms, and these harmful blooms have the potential to generate harmful toxins, which can detrimentally impact the aquatic environment and human health^2^. Consequently, it is imperative to identify a safe and efficient approach to combat algal blooms to safeguard the ecological safety of water. Currently, the primary techniques to control algal blooms and improve water quality include physical and chemical approaches and biological techniques^3^. The physical process is simple to operate, but it has the disadvantage of high cost^4^. Chemical approaches are often used for emergency treatment. However, it has the drawback of being environmentally unfriendly^5^. Therefore, biological approaches have been recommended to be useful in algal bloom control.

The allelopathy effect refers to the influences of secondary metabolites continuously released by plants to the environment during their growth processes on the growth of other organisms. Researchers have shown considerable interest in the allelopathic effects of aquatic plants, which emit chemical compounds into the water, on algae^6^. Such studies have important environmental and ecology implications^7,8^. Numerous aquatic plants have been identified as strong producers of allelochemicals^9,10^. Prior research has documented the effective suppression of *Microcystis aeruginosa* biomass by the *Landoltia punctate*, a floating herbaceous plant. Similar investigations have demonstrated that *Ceratophyllum demersum* and *Myriophyllum* aquaticum effectively prevent the growth of *M. aeruginosa*^7,11^. The above researches provide a safe and efficient approach to combat the growth of *M. aeruginosa*, a bloom-forming cyanobacteria, which could be used to prevent algal blooms to safeguard the ecological safety of water. Meanwhile, the allelopathic effects of *Stratiotes aloides* on the growth and antioxidative biomarkers of epiphytic and planktonic toxic cyanobacteria have been documented by previous research^12^. Although earlier research has established *Zanthoxylum bungeanum*'s potential allelopathy^13^, no information has been published on the impact of allelochemicals released by *Z. bungeanum* on the growth of *M. aeruginosa*. Accordingly, developing and utilizing allelochemicals released by *Z. bungeanum* to combat the growth of *M. aeruginosa* presents considerable research value.

*Z. bungeanum*, a Rutaceae family member, is widely cultivated in China, notably in Shandong, Hebei, Sichuan, Shanxi, and Gansu provinces^14^, and is employed as a spice for cooking attributed to its fresh aroma and taste^15^. Moreover, *Z. bungeanum* can also be widely used in nutraceutical and pharmaceutical industries due to its pharmacological activity^16^. At present, the utilization of *Z. bungeanum* resources mainly focuses on fruit harvesting and processing, neglecting the development and comprehensive utilization of various components in *Z. bungeanum*. Reports regarding the total flavonoids extracted from *Z. bungeanum* residue and their antioxidant activity are not currently available. Consequently, broadening the *Z. bungeanum* industry chain and encouraging the industry's diversification and sustainable growth is of great practical significance.

This study employed a four-variable to further optimize ultrasonic-assisted extraction of total flavonoids from *Z. bungeanum* residue and evaluate their antioxidant activities. Meanwhile, the feasibility of using allelopathic substances to inhibit *M. aeruginosa* growth was explored, and the photosynthetic inhibitory mechanism of *Z. bungeanum* residue extracts against *M. aeruginosa* was assessed. This study aims to achieve a "zero" surplus of *Z. bungeanum* residue resources, industrialize the resource utilization of *Z. bungeanum* waste, support the industry's healthy and sustainable growth, and comprehensively improve *Z. bungeanum*'s value.

## Materials and Methods

### Materials and reagents

The *Microcystis aeruginosa* strain (FACHB-315), obtained from the Freshwater Algae Culture Collection at the Institute of Hydrobiology (FACHB), was grown on a sterile BG-11 medium using autoclaved 250 mL Erlenmeyer flasks. The cultures were maintained in an intelligent light incubator at 25 ± 1 °C under a light intensity of 6000 lx with an automated 14 h/10 h light/dark interval. The microalgae were sub-cultured by renewing the medium every 7 days, and the stock cultures in the exponential growth stage were used for the experiments. *Z. bungeanum* residue was purchased from Chongqing Fuliang Grain and Oil Co., Ltd, China, which have permission to collect *Z. bungeanum*. And the acquisition of *Z. bungeanum* complies with relevant institutional, national, and international guidelines and legislation.

The rutin was acquired from Aladdin Holdings Group Co., Ltd, located in China. The compounds sodium nitrite (NaNO_2_) and aluminum nitrate (Al(NO_3_)_3_•9H_2_O) were acquired from Shanghai Macklin Biochemical Technology Co., Ltd, located in China. Ethanol was purchased from China National Medicines Corporation Ltd, China. Petroleum was purchased from Tianjin Fuyu Fine Chemical Co., Ltd, China. Sodium hydroxide (NaOH) was purchased from Xi’long Chemical Co., Ltd, China. All chemicals and solvents were of analytical grade unless otherwise specified.

### Preparation of extracts of* Z. bungeanum* residue

*Z. bungeanum* residue samples were crushed into powder and subjected to oven drying at a temperature of 60 °C until a stable weight was achieved. Then, the samples were filtered with a 140 mesh sieve and degreased with petroleum (7.5 mL petroleum/1.0 g sample). Subsequently, 6.5 g of degreased *Z. bungeanum* residue was placed in a conical flask, and then 130 mL of 60% ethanol was added. The mixtures were then incubated using an ultrasonic cleaning machine (JP-100S, Skymen Cleaning Equipment Shenzhen Co., Ltd, China) at the designated extraction temperature (80 °C), extraction time (55 min), and extraction power (300 W). The supernatant in the flask was harvested by vacuum filtration and was employed to determine the concentration of total flavonoids in the residue of *Z. bungeanum*. Finally, the supernatant was concentrated by Rotary Evaporator (SY-2000, Shanghai Yarong Biochemical Instrument Factory, China) to improve the flavonoid content, and the final concentration of total flavonoids from *Z. bungeanum* residue was 2.4 g/L. The experiment was conducted three times.

### Measurements of the content of *Z. bungeanum* residue total flavonoids

The content of *Z. bungeanum* residue total flavonoids was measured according to Li et al. (2019) with minor modifications^17^. Specifically, 0.2 mL extracts of *Z. bungeanum* residue were placed in a 5.0 mL centrifuge tube, and then 1.8 mL of 60% ethanol was added. Afterward, 0.12 mL of a solution containing 5% Sodium nitrite by mass was added, and the combination was kept for a duration of 6 min. Next, 0.12 mL of a solution containing 10% aluminum nitrate was added and allowed to sit for 6 min. Finally, the test solution was obtained by adding 1.6 mL of 4% Sodium hydroxide solution and 0.16 mL of deionized water. The final solution was mixed and allowed to stand for 15 min at 25 °C, and the absorbance was measured at 510 nm.

Rutin was used as a standard (Figure S1), and the relationship between concentrations of rutin and OD_510_ values was defined employing the following equation: Y = 11.23X + 0.0405 (R^2^ = 0.9929), where Y is the absorbance value (OD_510_); R^2^ is the correlation coefficient; and X is the concentrations of rutin (mg/mL). The content (mg/g) of *Z. bungeanum* residue total flavonoids was calculated using the following formula: Y = (C × V) / W × 100%, where C is the concentration of the test solution (mg/mL), V is the volume of the extraction solution (mL), and W is the sample weight (g).

### Single-factor experiment

#### Impact of liquid–solid ratio (LSR) on the content of *Z. bungeanum* residue total flavonoids

To investigate the impact of LSR on the content of *Z. bungeanum* residue total flavonoids, the LSR gradient ranged from 15:1 to 35:1 was explored. 0.1 g of degreased *Z. bungeanum* residue was placed in a 5.0 mL centrifuge tube with different volumes of 60% ethanol. The mixtures were then incubated at the designated extraction temperature (60 °C), extraction time (10 min), and extraction power (300 W), and the supernatant was harvested by centrifugation (12,000 rpm, 5 min). The extraction process was performed two times, and the supernatants were mixed to determine the concentration of total flavonoids in the residue of *Z. bungeanum*. The biological processes were replicated in three separate and concurrent studies.

#### Effect of extraction time on the content of*** Z. bungeanum ***residue total flavonoids

An investigation was conducted to assess the impact of different extraction time (5, 15, 25, 35, 45, 55, and 65 min) on the total flavonoids content of *Z. bungeanum* residue. 0.1 g of degreased *Z. bungeanum* residue was placed in a 5.0 mL centrifuge tube, and then 2.0 mL of 60% ethanol was added. The mixtures were then incubated at the designated extraction temperature (60 °C) and extraction power (300 W) with various of extraction time. The supernatant was collected by spinning it in a centrifuge and then utilized to determine the amount of total flavonoids present in the leftover *Z. bungeanum* material. The biological processes were replicated in three separate and concurrent studies.

#### Impact of the concentrations of ethanol on the content of*** Z. bungeanum ***residue total flavonoids

To study the impact of the concentrations of ethanol on the content of *Z. bungeanum* residue total flavonoids, 0.1 g degreased *Z. bungeanum* residue was placed in a 5.0 mL centrifuge tube, and then added 2.0 mL of various concentrations of ethanol (0%, 20%, 40%, 60%, 80%, 100%). The mixtures were then incubated at the designated extraction temperature (60 °C), extraction time (55 min), and extraction power (300 W), and the test solution was obtained using the same method as described above. The biological processes were replicated in three separate and concurrent studies.

#### Impact of ultrasonic temperature on the content of*** Z. bungeanum ***residue total flavonoids

In order to find the best ultrasonic temperature condition, optimization of ultrasonic temperature was regarded based on one factor at time assay. 0.1 g of degreased *Z. bungeanum* residue was placed in a 5.0 mL centrifuge tube, and then 2.0 mL of 60% ethanol was added. The mixtures were then incubated in the conical flask at the designated extraction time (55 min) and extraction power (300 W) with various extraction temperatures (40, 50, 60, 70, and 80 °C), and the test solution was obtained using the same method as described above.

### Evaluation of *Z. bungeanum* residue total flavonoids antioxidant activities

Total oxidant capacity was measured using assay kits (Cat. No. A015-1-2, Nanjing Jiancheng Bioengineering Insitute, China) according to the manufacturers’ recommendations. The absorbance was measured at 520 nm, and an increase of 0.01 in the OD_520_ value at 1 min with 37 °C by 1 mL extract sample was recorded as one unit of total antioxidant capacity. The biological processes were replicated in three separate and concurrent studies.

### Microalgae Growth Inhibition Experiment

*M. aeruginosa* with the same number of cells at the start was subjected to *Z. bungeanum* residue extracts at concentrations of 5.0 and 10.0 mg/L. This was done in a controlled laboratory setting, using 250 mL conical flasks containing 50 mL of BG-11 culture medium. The experimental conditions were the same as those described in “Materials and reagents”, and each culture was sealed with a membrane. The control was conducted without using any *Z. bungeanum* residue extracts in order to establish the baseline growth. An analysis was conducted to assess the *M. aeruginosa* growth by examining the impact of various doses of *Z. bungeanum* residue extracts on the photosynthetic inhibitory mechanism of the organism. The Aquapen system (AquaPen-C AP110-C) was used to measure the chlorophyll fluorescence in cultures with the same cell density. To maintain the PSII reaction centers in a fully open state, all samples were subjected to a 30-min period of darkness prior to testing. The OJIP transient exhibited a saturated light intensity of 2100 µmol/m^2^/s. The abbreviations, equations, and meanings of the JIP-test parameters are outlined in accordance with Cheng et al.^18^. The experiment was carried out over a period of 0, 1, and 3 h, with each experiment being repeated three times. Moreover, microscopic photography technology was used to reflect the number of algal cells, and an oxidative stress test was carried out using the total antioxidant activity kit to investigate free radical levels, which could provide valuable insights into the potential oxidative stress induced by the *Z. bungeanum* residue extracts.

### Statistical analysis

The data underwent univariate analysis of variance (ANOVA) and were then subjected to Duncan's multiple comparisons tests. Statistical significance was determined at a significance level of 5% (P < 0.05). The tests were conducted using the SPSS 19.0 statistical software (SPSS Inc., Chicago, USA), and the figures were generated using GraphPad Prism 9.0 software (San Diego, CA, USA), in which all tests were measured in triplicate, and results were expressed as mean ± standard deviation.

## Results and discussion

### Optimisation of ultrasonic-assisted extraction of *Z. bungeanum* residue total flavonoids

**Figure 1:**
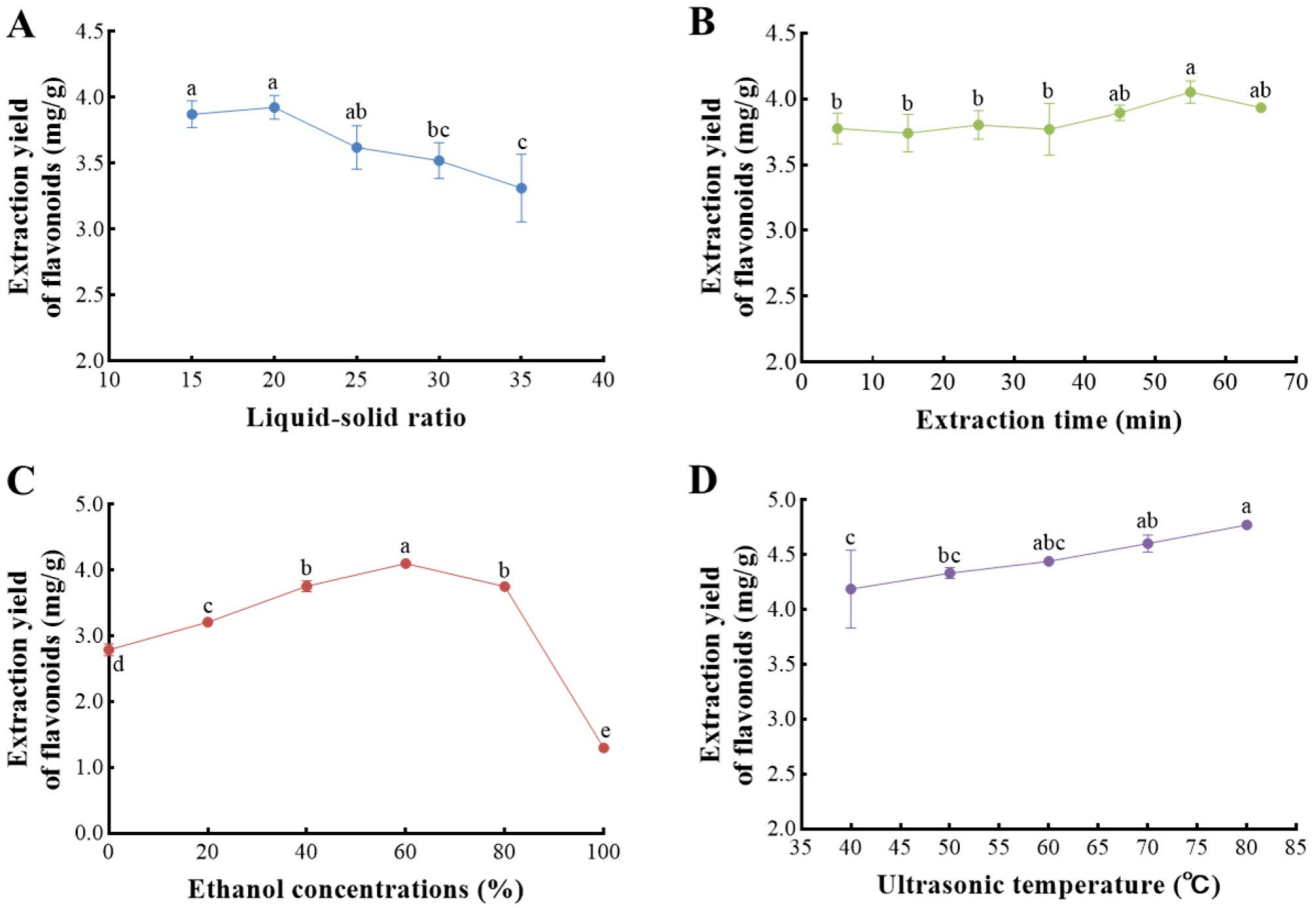
Effects of four independent variables on the extraction yield of *Z. bungeanum* residue total flavonoids. (**A**) liquid–solid ratio; (**B**) extraction time; (**C**) ethanol concentrations; (**D**) ultrasonic temperature. Values represent the mean of three independent measurements (n = 3) and bars indicate SD.

As shown in Fig. 1, the single-factor experiment was designed to optimize the ultrasonic-assisted extraction process of *Z. bungeanum* residue total flavonoids, and four factors, such as LSR, extraction time, ethanol concentrations, and ultrasonic temperature, have been chosen to investigate the effect on the total flavonoids content.

#### Effect of LSR on the extraction yield of*** Z. bungeanum*** residue total flavonoids

Previous studies have confirmed that the LSR played an important role in improving the efficiency of flavonoid extraction^19^. In this study, an experimental investigation was conducted with an LSR ranging from 15 to 35 mL/g while maintaining a fixed extraction temperature of 60 °C and an extraction period of 10 min, ethanol concentration of 60%, and extraction power to 300 W. The LSR had an effect on *Z. bungeanum* residue total flavonoids, and increasing in the tested LSR (from 15 mL/g to 20 mL/g) could improve the total flavonoids content (Fig. 1A). The increased LSR contributes to improving the contact area between *Z. bungeanum* residue and the extraction solvent, resulting in the rapid release of flavonoids^20,21^. Meanwhile, the increase of the total flavonoids content leveled off at a ratio of 20 mL/g, indicating the increase of LSR can also lead to a slowdown in the accumulation rate of flavonoids^22^. However, the total flavonoids content showed a sharp downward trend when the LSR varied from 20 mL/g to 35 mL/g, which was due to the large differences between active constituent concentration in the material and solvent boundary layer and big active diffusion force with the increment of LSR^21^. A similar result was previously reported in Silva et al. and Wen and Mai^23,24^. Consequently, the LSR for extraction of *Z. bungeanum* residue total flavonoids was selected at 20 mL/g.

#### Impact of extraction time on the extraction yield of*** Z. bungeanum*** residue total flavonoids

By setting extraction time from 5 to 65 min, the effect of extraction time on the *Z. bungeanum* residue total flavonoids was tested while other extraction parameters were kept constant (Fig. 1B). There was no significant difference in the extraction yield of total flavonoids from *Z. bungeanum* residue (P > 0.05), but it started to increase in 35 min. When the extraction time was less than 55 min, the extraction yield increased rapidly. This is because the flavonoids in *Z. bungeanum* residue are gradually released as the extraction time prolongs. However, the extraction yield of total flavonoids from *Z. bungeanum* residue decreased slightly with the further increase in extraction time due to the flavonoids’ structure change and their oxidation^25^. Therefore, the optimal suitable extraction time for the extraction of *Z. bungeanum* residue total flavonoids compounds was set to 55 min.

#### Impact of ethanol concentrations on the extraction yield of* Z. bungeanum *residue total flavonoids

The impact of various ethanol concentrations on the extraction of *Z. bungeanum* residue total flavonoids compounds is shown in Fig. 1C. Apparently, the total flavonoids content was promoted rapidly when the ethanol concentrations ranged from 0 to 60%. Nevertheless, the yield of total flavonoids extraction exhibited a declining pattern as the ethanol concentration was above 60%. The extraction efficiency of active ingredients was significantly influenced by the polarity of the extraction solvent and the solubility of active compounds^25,26^. Generally speaking, polar flavonoid compounds have a higher solubility in low-concentration ethanol, and greater concentration ethanol is beneficial for the extraction of non-polar flavonoid compounds^27^. In this study, when the ethanol concentrations ranged from 0 to 60%, the solubility of flavonoids gradually increased. Other substances with smaller polarity may be dissolved together first when the ethanol concentrations exceed 60%, contributing to a hindrance in the extraction efficiency of *Z. bungeanum* residue total flavonoid^28^. Consequently, an optimal ethanol concentration of 60% was favorable for *Z. bungeanum* residue total flavonoid production.

#### Impact of ultrasonic temperature on the extraction yield of*** Z. bungeanum ***residue total flavonoid

By fixing LSR to 20 mL/g, ethanol concentration to 60%, and extraction time to 55 min, the impact of extraction temperature (40, 50, 60, 70, and 80℃) on the *Z. bungeanum* residue total flavonoid was investigated (Fig. 1D). In this study, the extraction yields of *Z. bungeanum* residue total flavonoid significantly elevated as the ultrasonic temperature increased from 40 to 80 °C. This was because elevating ultrasonic temperature helped to enhance the diffusion coefficients and solubility of flavonoids. A similar result was previously reported in Liu et al.^27^. However, higher temperature treatment would affect the composition and bioactivity of total flavonoid. Thus, 80 °C was selected as the optimal extraction temperature.

Based on the findings from the single factor tests mentioned above, a Central Composite design (CCD) of response surface methodology (RSM) was used to maximize the extraction efficiency of *Z. bungeanum* residue total flavonoid. This design included three levels and four variables. Surprisingly, the total flavonoid extraction yield from *Z. bungeanum* residue was lower than that by single-factor experiments (Date not shown). Consequently, optimum extraction conditions were LSR of 20 mL/g, solvent concentration of 60%, extraction time of 55 min, and ultrasonic temperature of 80 °C. Under these conditions, extracts of *Z. bungeanum* residue were prepared for subsequent toxicological experiments, and the initial concentration of total flavonoid from *Z. bungeanum* residue was 2.4 g/L.

### Evaluation of *Z. bungeanum* residue total flavonoids antioxidant activities

**Figure 2:**
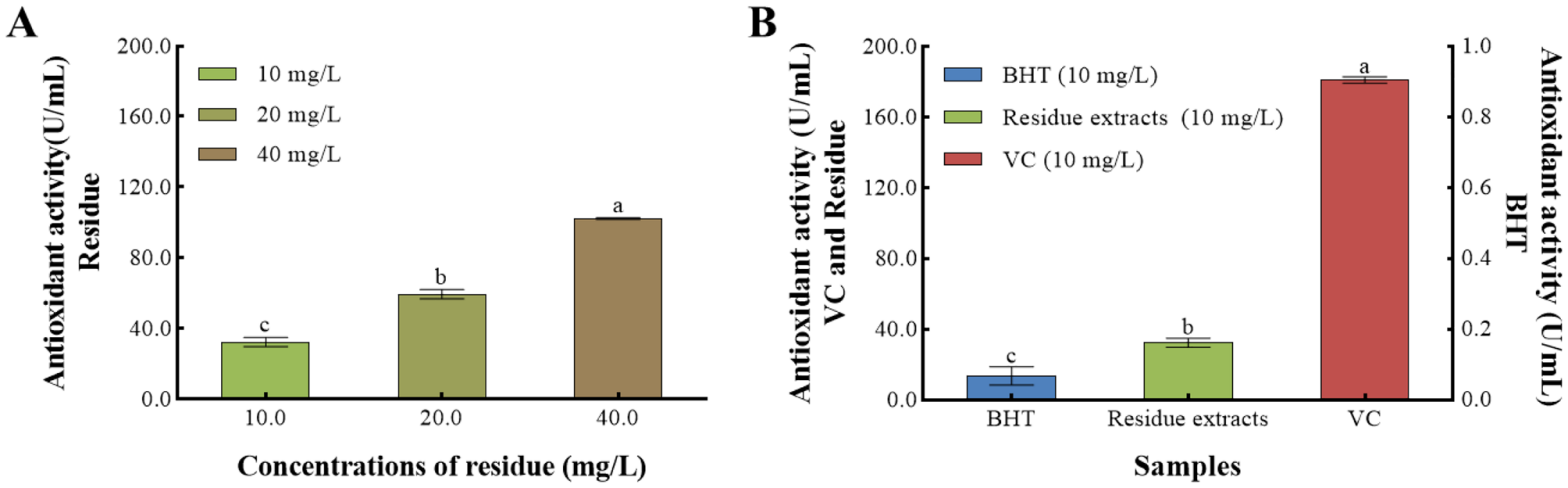
Comparison of total oxidant capacity of *Z. bungeanum* residue extracts, Butylated hydroxytoluene, and Vitamin C. Values represent the mean of three independent measurements (n = 3) and bars indicate SD. Different letters indicate a significant difference at the 0.05 level (P < 0.05, Duncan's multiple range test).

Total oxidant capacity was used for measuring the *Z. bungeanum* residue total flavonoids antioxidant capacity. In this study, the total oxidant capacity of *Z. bungeanum* residue extracts, Butylated hydroxytoluene (BHT), and Vitamin C (VC) were assessed (Fig. 2). As shown in Fig. 2A, the total oxidant capacity of *Z. bungeanum* residue extracts showed dose-dependent effects when the concentration of *Z. bungeanum* residue total flavonoids was in the range of 10–40 mg/mL, and the total oxidant capacity at 40 mg/mL was 3.16-fold higher than that at 10 mg/mL (Fig. 2A). Meanwhile, total oxidant capacity was measured for different samples of 10 mg/mL (Fig. 2B), and the enzyme activities of *Z. bungeanum* residue extracts, Butylated hydroxytoluene (BHT), and Vitamin C (VC) were determined to be 32.354, 0.068, and 181.053 U/mL, indicating the antioxidant capacity of *Z. bungeanum* residue extracts was between BHT and VC.

### Fast chlorophyll fluorescence transient (OJIP) is an effective tool to evaluate the growth of *M. aeruginosa* exposed to *Z. bungeanum* residue extracts

*M. aeruginosa* was cultured in different *Z. bungeanum* residue extract concentrations to investigate the effect of *Z. bungeanum* residue extracts stress on cell growth, and the cell density significantly decreased as the *Z. bungeanum* residue extract concentrations increased (Figure S2). Meanwhile, an analysis was conducted to assess the *M. aeruginosa* growth by examining the impact of various doses of *Z. bungeanum* residue extracts on the photosynthetic inhibitory mechanism of *M. aeruginosa*. Photosynthesis is a highly responsive physiological process that may be affected by changes in the environment. Among the several components of the photosynthetic electron transport chain, Photosystem II (PSII) is particularly susceptible to environmental challenges in algae^29,30^. The chlorophyll fluorescence transient curve is often used to indicate changes in the principal photochemical process and the electron transfer state^31^. Accordingly, Chlorophyll fluorescence transient was used as an effective tool to evaluate the growth of *M. aeruginosa* exposed to *Z. bungeanum* residue extracts.

#### OJIP fluorescence transient analysis

The photosynthetic efficiency of *Z. bungeanum* residue extracts-treated *M. aeruginosa* was further examined using the Aquapen system, and the fast chlorophyll fluorescence transient curves (OJIP curves) of three *Z. bungeanum* residue extract levels were compared. When *M. aeruginosa* was dark-adapted, the photosynthetic electron transport chain was in a quiescent condition, and the quantum yield was at its minimum level. When the lightning took place, most of the electrons generated by water photolysis reduced the Q_A_ molecules, and the quantum yield gradually rose to the J level. Subsequently, Q_B_ molecules are similarly reduced to form the I phase. Finally, fluorescence quantum yield reaches the highest level when the PQ pool has reached its peak of reduction^32^.

**Figure 3:**
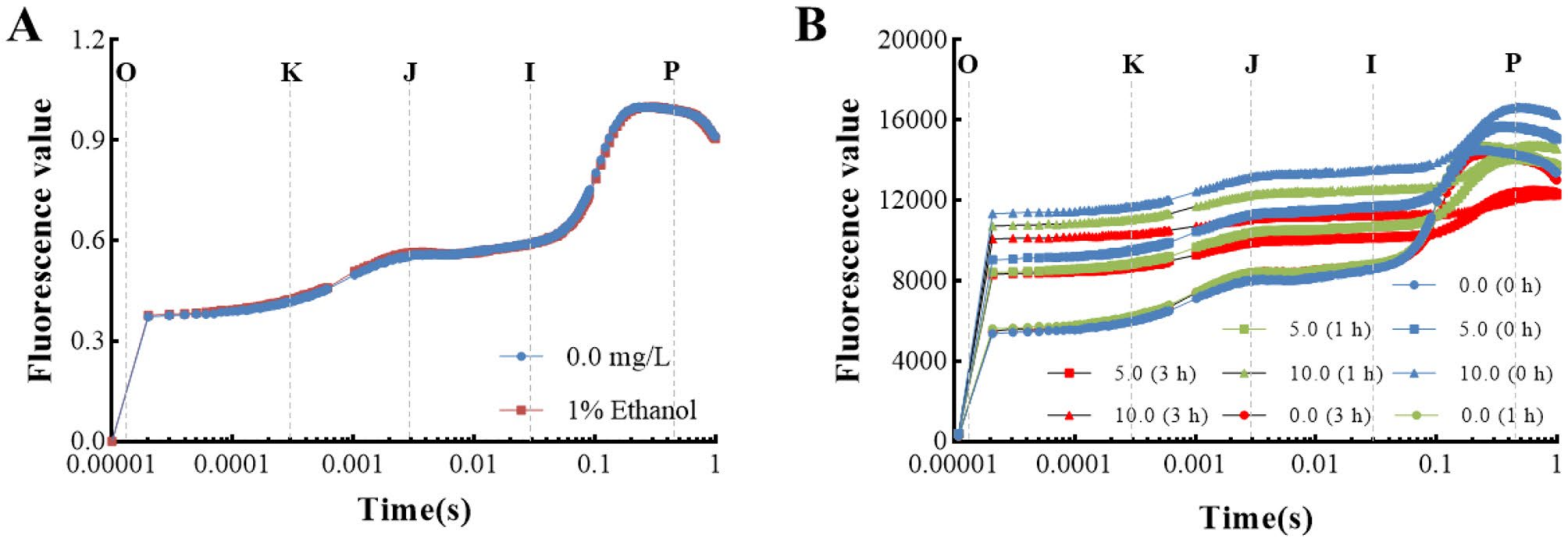
Chlorophyll a fluorescence OJIP transient curves of *M. aeruginosa* exposed to (**A**) 1% Ethanol and (**B**) three *Z. bungeanum* residue extract levels for 1 h. The transients are plotted on a logarithmic time scale. The marks indicate the time points used in the JIP-test for the calculation of structural and functional parameters. The signals are the fluorescence intensity O (at 20 μs), K (at 300 μs), J (at 2 ms), I (at 30 ms), and P (at the time of the maximal fluorescence intensity). Values represent the mean of three independent measurements (n = 3).

In this study, 60% ethanol was used to extract *Z. bungeanum* residue total flavonoids, and the initial concentration of total flavonoids from *Z. bungeanum* residue was 2.4 g/L under the optimal conditions. *M. aeruginosa* with equal initial cell densities was exposed to *Z. bungeanum* residue extracts in concentration gradients of 5.0, 10.0, and 15.0 mg/L, and the ethanol concentration in the culture medium at all treatment groups was less than 1%. Consequently, the effect of 1% ethanol on *M. aeruginosa* was studied. Figure 3A displays the chlorophyll fluorescence kinetic curves of *M. aeruginosa* subjected to 1% ethanol at 1 h. There was no significant difference in chlorophyll fluorescence transient curve among *M. aeruginosa* cells cultured at 1% ethanol and without ethanol, indicating 1% ethanol had no significant effect on the growth of *M. aeruginosa*.

OJIP curves of *M. aeruginosa* subjected to different concentrations of *Z. bungeanum* residue extracts are shown in Fig. 3B. The variations in different concentrations of *Z. bungeanum* residue extracts in *M. aeruginosa* were reflected in the OJIP fluorescence transients. The results obtained show that the presence of *Z. bungeanum* residue extracts had a significant impact on the fluorescence transients, with the transients progressively increasing as the concentration of *Z. bungeanum* residue extracts rose. The rise of the O–J part of the fluorescence indicates some PSII reaction centers were closed, and the electrons on the acceptor side of the PSII reaction center decreased due to the reduction of Q_A_ molecules in the photosystem II^29,33^. The rise of the J–I part of the fluorescence is related to the inhibition of downstream electronic receptors of Q_A_, such as Q_B_ molecules, cytochrome b6f., and plastocyanin^34^. In order to assess the photosynthesis damage under *Z. bungeanum* residue extracts stress, the JIP-test was employed to further identify sensitive functions, according to Strasser and Stirbet^35^, including energy capture, energy distribution, electron transport, etc. Also, the meaning of relevant measurement parameters has been recorded in previous literature^29^.

#### ***Z. bungeanum ***residue extracts damaged the oxygen-evolving complex structure

**Figure 4:**
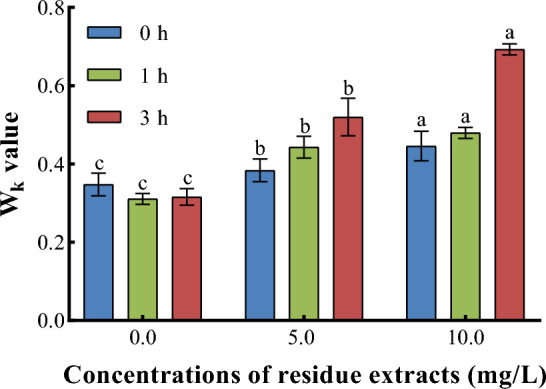
W_k_ value of *M. aeruginosa* exposed to three *Z. bungeanum* residue extract levels. Values represent the mean of three independent measurements (n = 3) and bars indicate SD. Different letters indicate a significant difference at the 0.05 level (P < 0.05, Duncan's multiple range test).

As shown in Fig. 4, the effect of exposing *M. aeruginosa* to elevated concentrations of *Z. bungeanum* residue extracts on the donor side of the PSII reaction center was evaluated as a W_k_ value. W_k_ parameter was used to reflect the change of the K point in the OJIP curve and analyze damage to the photosynthetic apparatus^29^. In this study, the inhibition effect of *Z. bungeanum* residue extracts was apparent immediately at 3 h, and the most pronounced increase in W_k_ value was seen in 10 mg/L, which was significantly higher (P < 0.01) than that of the control, indicating that *Z. bungeanum* residue extracts could damage the oxygen-evolving complex structure and result in photosynthetic electron transport disorder in PSII.

#### *Z. bungeanum* residue extracts influenced the energy capture and distribution of PSII

**Figure 5:**
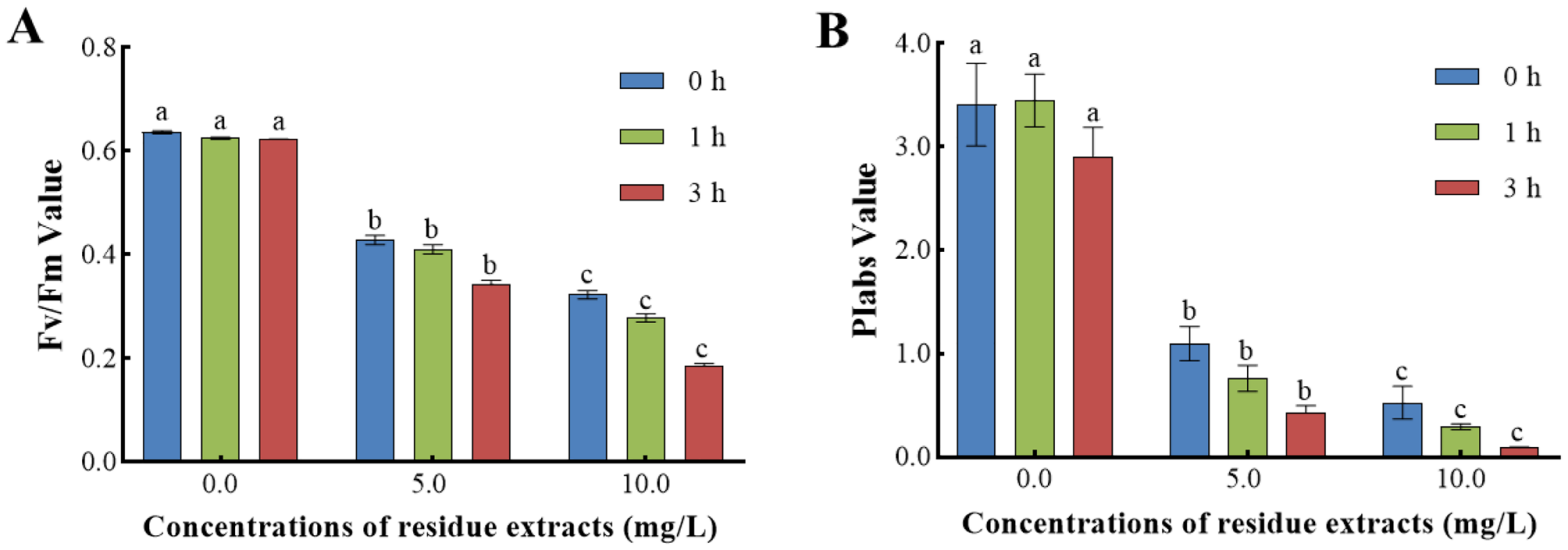
*Z. bungeanum* residue extracts influenced the energy capture of PSII. (**A**) Ratio of variable to maximum fluorescence (Fv/Fm) and (**B**) performance index on absorption basis (PIabs) under different *Z. bungeanum* residue extract treatments. Values represent the mean of three independent measurements (n = 3) and bars indicate SE. Different letters indicate a significant difference at the 0.05 level (P < 0.05, Duncan's multiple range test).

We determined the impact of various *Z. bungeanum* residue extracts on the energy capture of PSII in *M. aeruginosa* (Fig. 5). We observed substantial variations in the Fv/Fm values and PIabs values of samples treated with various *Z. bungeanum* residue extracts (P < 0.01). Opposed to the control group, the Fv/Fm values of each *Z. bungeanum* residue extract treatment decreased to varying degrees, with the smallest decrease observed under 5 mg/L and the largest decrease observed under 10 mg/L (Fig. 5A). A similar trend has been obtained in the performance index PIabs (Fig. 5B), indicating that the PSII reaction center's overall performance had been damaged^36^. It is worth noting that Fv/Fm and PIabs values were reduced by 69.98% and 96.90%, respectively, in cells treated with 10 mg/L *Z. bungeanum* residue extracts compared to cells without *Z. bungeanum* residue extracts in the media at 3 h. This finding indicates that PIabs exhibited more sensitivity than Fv/Fm towards *Z. bungeanum* residue extracts, which aligns with earlier studies^29,37^.

**Figure 6:**
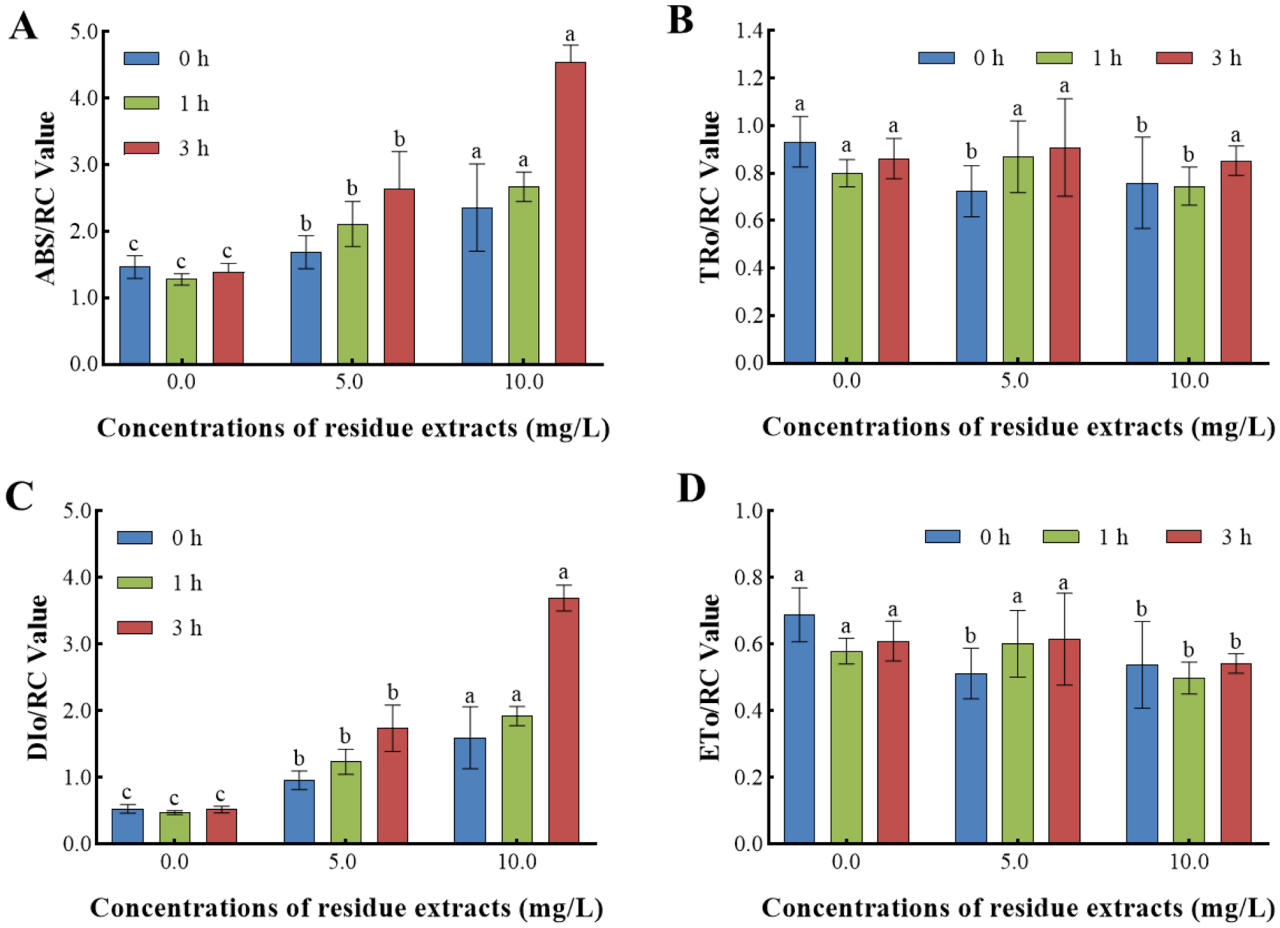
*Z. bungeanum* residue extracts influenced the energy distribution of PSII. Chlorophyll fluorescence parameters of the reaction center of *M. aeruginosa* cultured in the presence of different *Z. bungeanum* residue extract concentrations. Values represent the mean of three independent measurements (n = 3) and bars indicate SE. Different letters indicate a significant difference at the 0.05 level (P < 0.05, Duncan's multiple range test).

We next determined the implications of various *Z. bungeanum* residue extracts on the energy distribution of PSII in *M. aeruginosa* (Fig. 6). An increase in *Z. bungeanum* residue extract concentration contributed to an elevation in ABS/RC and DIo/RC (Fig. 6A–C). Specifically, the ABS/RC and DIo/RC values were 2.29-fold and 6.08-fold higher than that of the control, as 10 mg/L *Z. bungeanum* residue extracts were treated, indicating a significant stimulation (P < 0.01). The rise in ABS/RC signifies a decrease in the proportion of active reaction centers^35^. Instead, the decline of ETo/RC in response to *Z. bungeanum* residue extracts stress was obtained, and the ETo/RC as 10 mg/L *Z. bungeanum* residue extracts was 10.98% lower than that of the control lacking *Z. bungeanum* residue extracts at 3 h (Fig. 6D). In the present study, the decrease in quantum yield of photochemical energy conversion and electronic transfer efficiency was observed in *M. aeruginosa* after exposure to *Z. bungeanum* residue extracts, as evidenced by the decrease in Fv/Fm and ETo/RC. Previous studies have confirmed that increasing flavonoid concentration caused a significant reduction in the Fv/Fm, which was consistent with our research results of this study^38^. Meanwhile, more light energy was dissipated into heat and fluorescence, and heat dissipation is essential for protecting *M. aeruginosa* from stress-induced damage, which may be a self-protective mechanism in *M. aeruginosa*.

#### Z. bungeanum residue extracts inhibited the *electron* transport of PSII

**Figure 7:**
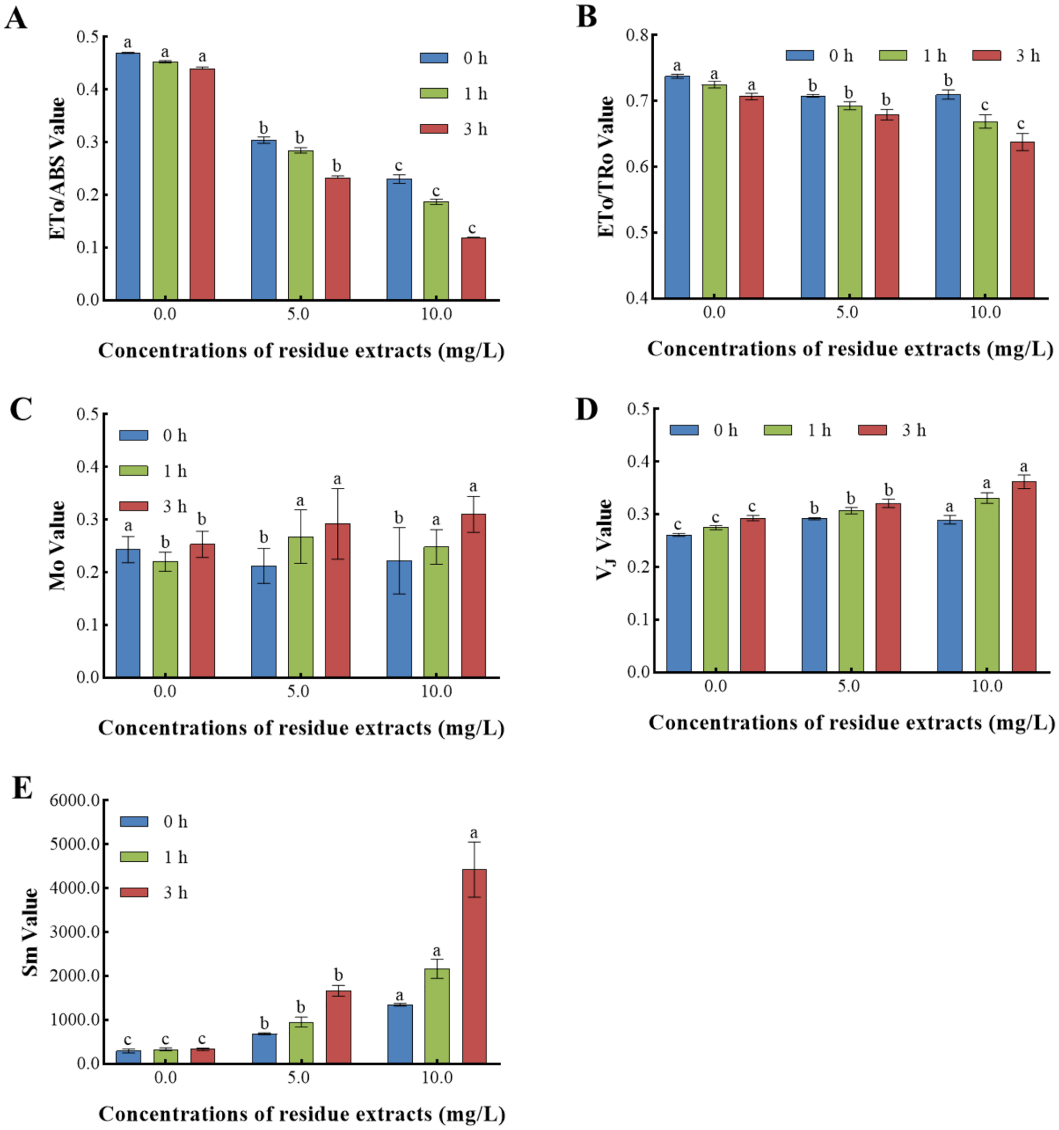
*Z. bungeanum* residue extracts inhibited the electron transport of PSII. Values represent the mean of three independent measurements (n = 3) and bars indicate SD. Different letters indicate a significant difference at the 0.05 level (P < 0.05, Duncan's multiple range test).

The impact of various *Z. bungeanum* residue extract concentrations on the electron transport of the PSII reaction center of *M. aeruginosa* has been investigated (Fig. 7). ETo/ABS and ETo/TRo represented the photosynthetic electron transport efficiency of PSII^39^. The ETo/ABS and ETo/TRo values in the 10 mg/L group were decreased by 72.95% and 9.76% as opposed to the control group at 3 h, respectively (Fig. 7A,B), and the decrease of ETo/TRo parameter indicated a susceptibility to photo-inhibition, which can be further confirmed by the increase in DIo/RC value^40^. In addition, there was a significant discrepancy in the Mo, V_J_, and Sm values (P < 0.01) seen among *M. aeruginosa* cells cultivated at varying doses of *Z. bungeanum* residue extracts (Fig. 7C–E), and the increase in Mo, V_J,_ and Sm values indicated the limited electron flux beyond Q_A_, resulting in a blockade of electron transport of PSII^41^.

### Evaluation of the potential oxidative stress induced by the *Z. bungeanum* residue extracts in *M. aeruginosa*

**Figure 8:**
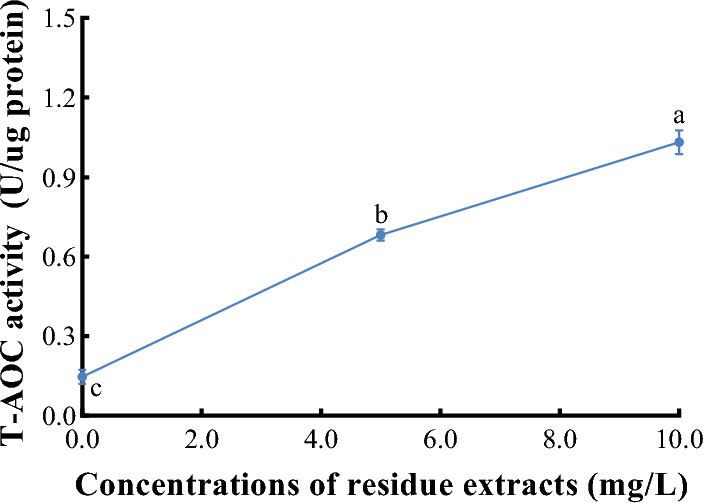
Comparison of total oxidant capacity of *M. aeruginosa* cells under different *Z. bungeanum residue* extract concentrations. Values represent the mean of three independent measurements (n = 3) and bars indicate SD. Different letters indicate a significant difference at the 0.05 level (P < 0.05, Duncan's multiple range test).

We also examined the effect of different *Z. bungeanum* residue extract concentrations on the total antioxidant (T-AOC) activity of *M. aeruginosa* cells (Fig. 8). There was a significant difference in T-AOC activity (P < 0.05) among *M. aeruginosa* cells cultured at different concentrations of *Z. bungeanum* residue extract. The T-AOC activity increased with an increase in *Z. bungeanum* residue extract concentrations. When treated with 10 mg/L *Z. bungeanum* residue extract, the T-AOC activity was 6.02-fold higher than that of the control, indicating a significant increase relative to untreated cells (P < 0.01). In the current investigation, the accumulation of reactive oxygen species (ROS) in cells subjected to *Z. bungeanum* residue extract-induced stress indicates cellular damage, prompting the activation of cellular antioxidant defenses to mitigate ROS levels. The elevated T-AOC observed in treated cells suggests the presence of a robust antioxidant system that effectively protects against oxidative stress and suppresses ROS production in response to *Z. bungeanum* residue extract-induced stress. Furthermore, the enhanced capacity for ROS detoxification enables treated cells to sustain their photosynthetic activity.

## Conclusions

To sum up, the extraction process of total favonoids from *Z. bungeanum* residue and its antioxidant activities were investigated, and the optimum extraction conditions of total favonoids from *Z. bungeanum* residue were LSR of 20 mL/g, solvent concentration of 60%, extraction time of 55 min, and ultrasonic temperature of 80 °C. Meanwhile, the photosynthetic inhibitory mechanism of *Z. bungeanum* residue extracts against *M. aeruginosa* was assessed, and the toxicity effect was concentration-dependent. *Z. bungeanum* residue extracts damaged the oxygen-evolving complex structure, influenced energy capture and distribution, and inhibited the electron transport of PSII in *M. aeruginosa*. Furthermore, the enhanced capacity for ROS detoxification enables treated cells to sustain their photosynthetic activity. The allelopathic mechanism of *Z. bungeanum* residue extracts revealed in this study can be used as theoretical evidence for the development of allelopathic algae-inhibiting agents, thus providing insight into the ecological management of cyanobacterial blooms.

### Supplementary Information

Supplementary Information.

## Data Availability

All relevant data are within the manuscript, and the data are available from the corresponding author on reasonable request.
