# Supplementary material for: Ultrasonic-assisted extraction of total flavonoids from Zanthoxylum bungeanum residue and their allelopathic mechanism on Microcystis aeruginosa
Source: Sci Rep. 2024 Jun 8;14:13192. doi: 10.1038/s41598-024-64129-x (PMC11162473; doi:10.1038/s41598-024-64129-x)

**Ultrasonic-assisted extraction of total flavonoids from *Zanthoxylum bungeanum* residue and their allelopathic mechanism on *Microcystis aeruginosa***

Jie Cheng ^1,3,†,^*, Chengshuai Xu ^1,†^, Yang Sun ^1,†^, Qiuhan Yu ^1^, Shuo Ding ^1^, Yucai Wang ^1^, Wenxue Wei ^2^, Wei Xu ^1^, Chaobo Zhang ^1,4,^*, Donghui Gong ^2,^*

^1^ School of Life Sciences, Liaocheng University, Liaocheng, 252000, China;

^2^ School of Life Science and Technology, Inner Mongolia University of Science and Technology, Baotou 014010, China;

^3^ Shandong Sanduha Ecological Agriculture Technology Co., Ltd, Liaocheng, 252000, China;

^4^ Shandong Nongmanyi Agricultural Technology Co., Ltd, Liaocheng, 252000, China.

^†^ These authors contributed equally to this work.

* Corresponding authors:

chengjie@lcu.edu.cn (J.C.); zhangchaobo@lcu.edu.cn (C.Z.); gongdh1976@163.com (D.G.)

**Figure legend**

**Figure S1** The relationship between concentrations of rutin and OD_510_ values. Values represent the mean of three independent measurements (n = 3).

**Figure S2** The cell density of *M. aeruginosa* cells under different *Z. bungeanum residue* extract concentrations. Microscopic photography technology was used to reflect the number of algal cells.

**Figure S1**

**Figure S2**

**
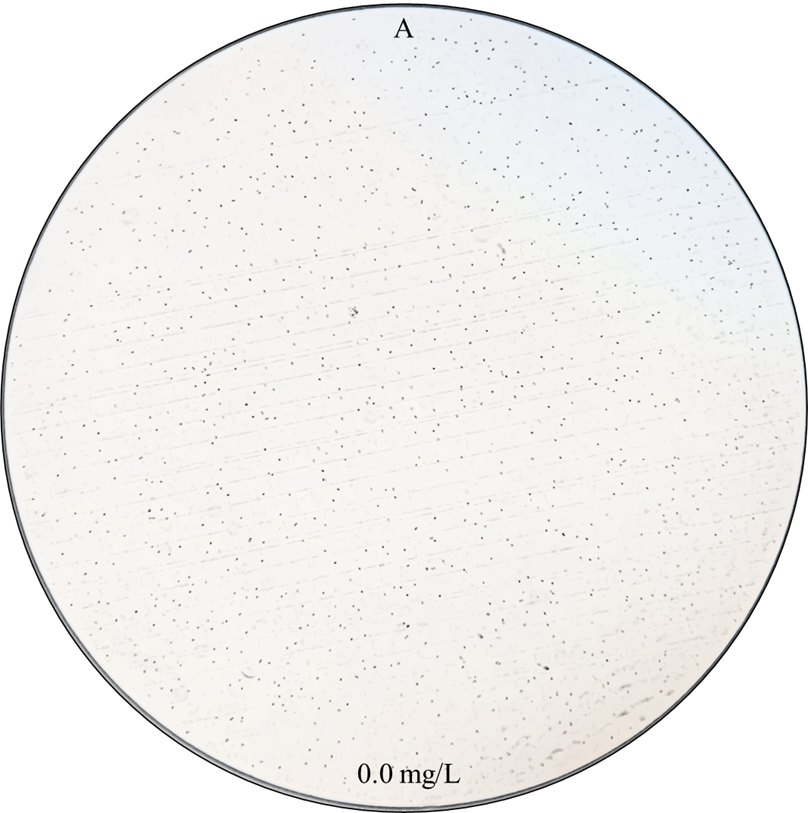
**

**
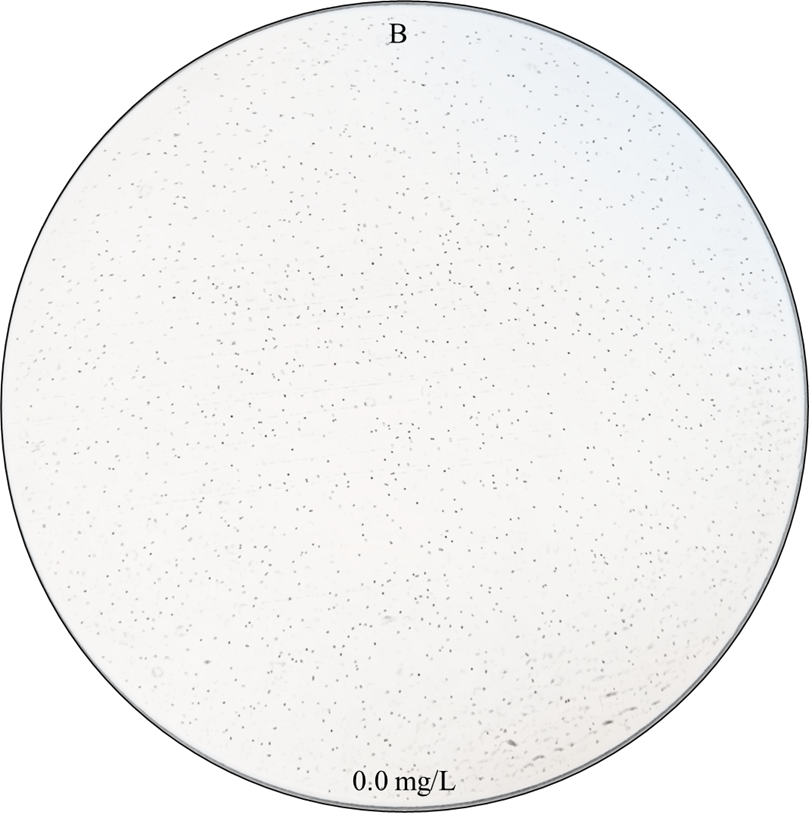
**


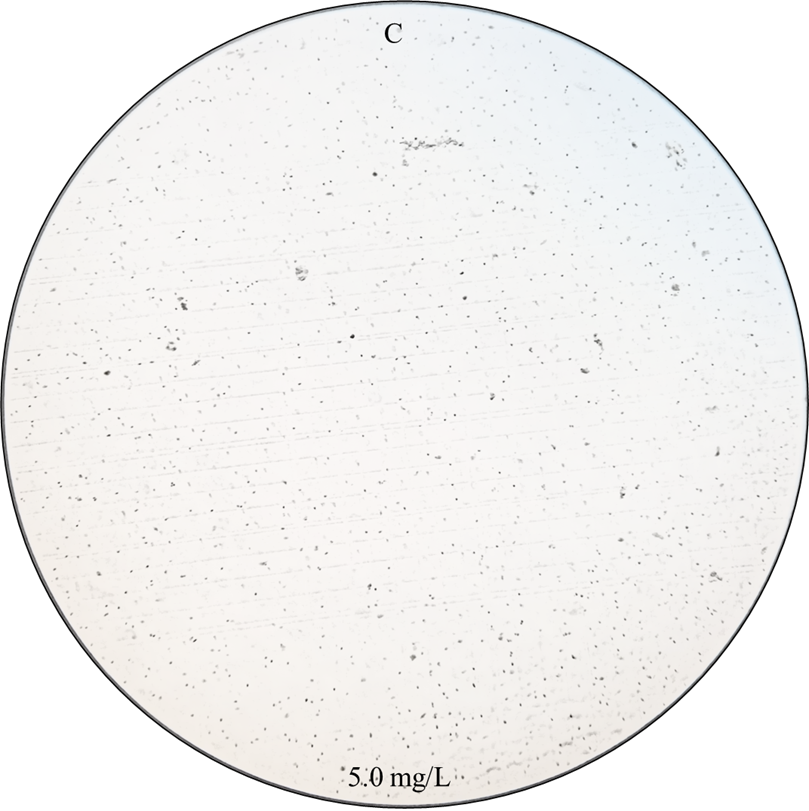


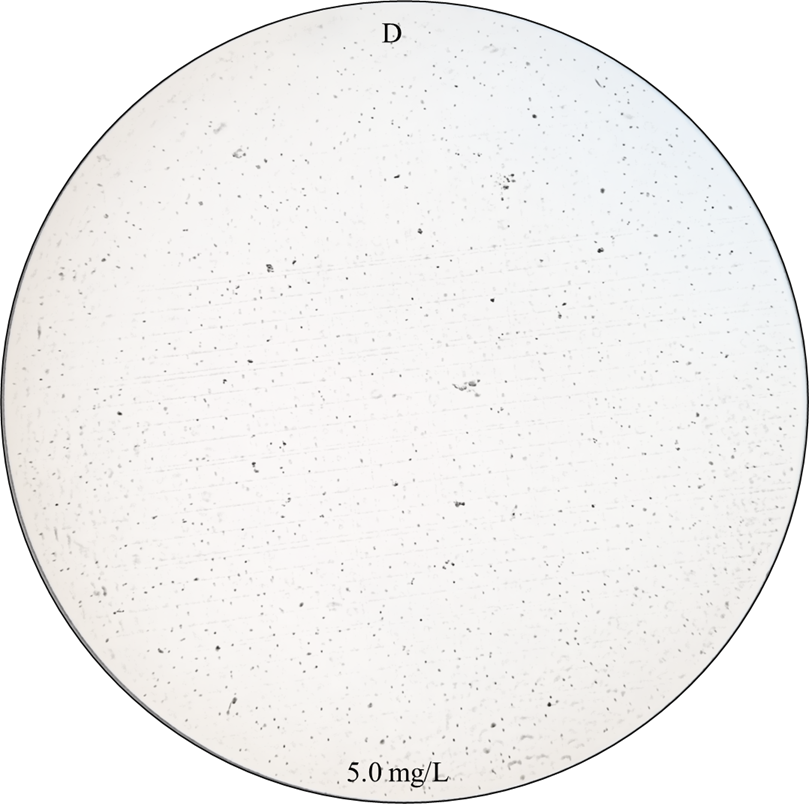


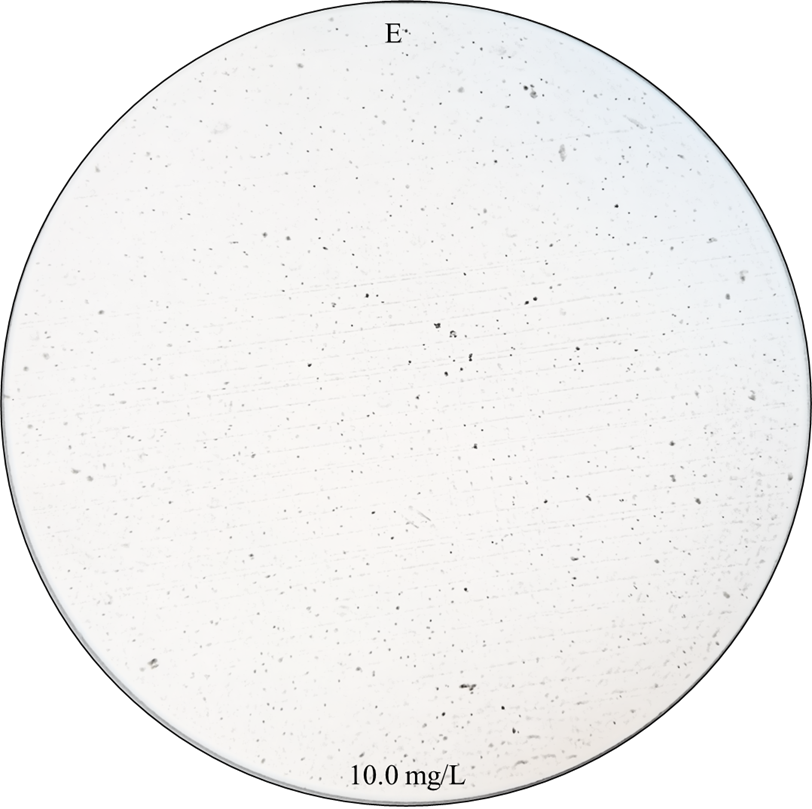


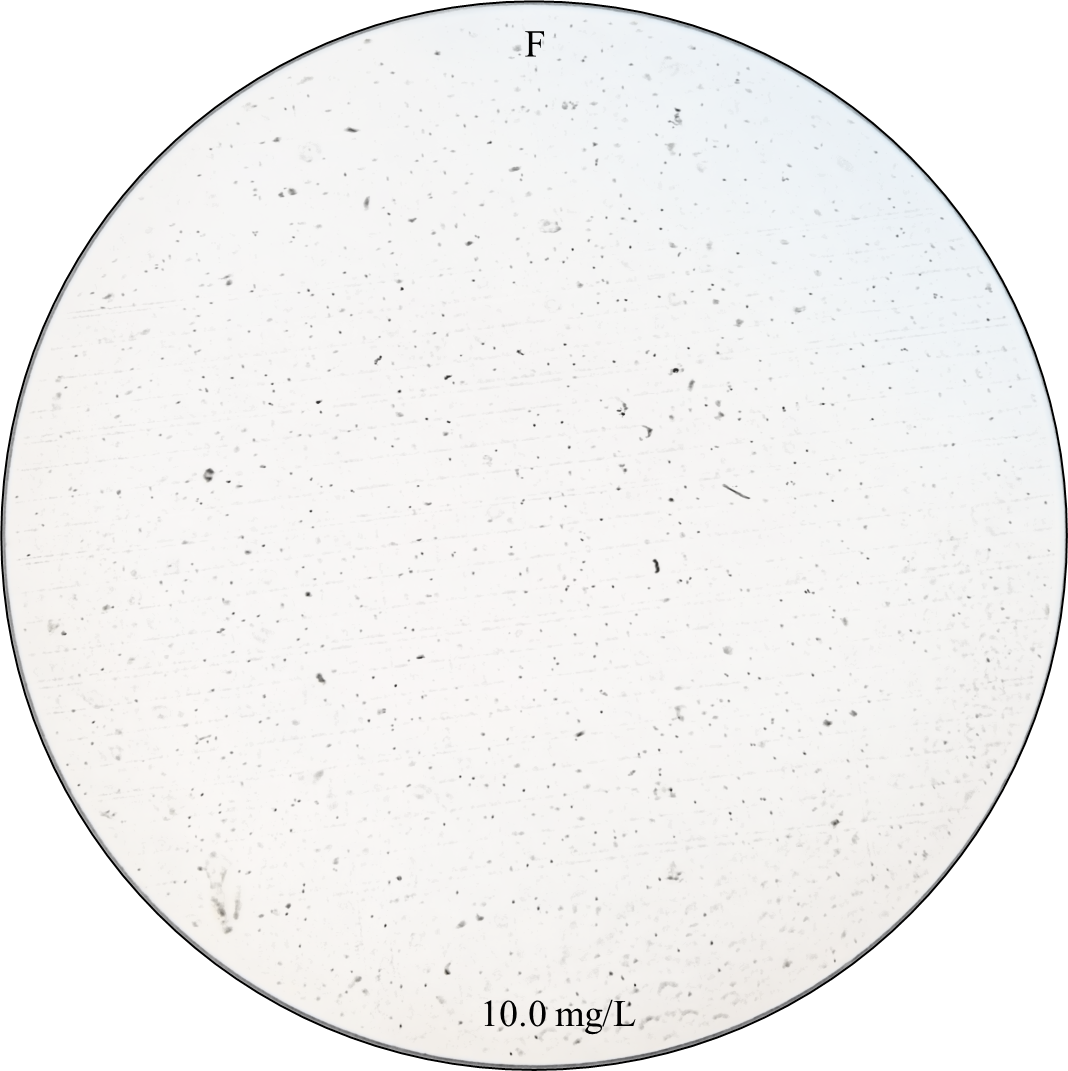

Supplement: Supplementary file 1 — Supplementary Information. [file 41598_2024_64129_MOESM1_ESM.docx]
